# Doctor-patient relationships, laws, clinical guidelines, best practices, evidence-based medicine, medical errors and patient safety

**Published:** 2013-03-31

**Authors:** Claudio Violato

**Affiliations:** University of Calgary

Patient safety has now become a mantra of modern medical practice. Rules, laws, guidelines, evidence and best practices are frequently invoked to improve patient safety. These are not new; they have governed the practice of medicine since antiquity.

A set of laws, known as the Code of Hammurabi (circa 1740 B.C.E.) have come down to us from the Babylonians after its namesake, the founder of the Babylonian empire.^1^ These 282 statues or common laws governed nearly all aspects of social, political, economic and professional life including those pertaining to physicians, surgeons, veterinarians, midwifes and wet nurses. Carefully conscribed details were devoted to specifying the relationship between patients and practitioners, including fees and penalties. Problems of “internal medicine” were dealt with physicians of the priestly class who saw to internal disorders caused by supernatural factors.

The surgeon who dealt with physical problems, however, was accountable for both remuneration and liability to earthly courts. If a doctor performed surgery, generally with a bronze knife, and saved the life or eyesight of an upper class citizen, he was to be paid 10 shekels of silver. A similar outcome for a commoner was worth 5 shekels and only 2 shekels for a slave. If the outcome for the upper class citizen was bad (blindness or death), the doctor’s hand was amputated. If a slave died because of the surgery, the doctor had to provide a replacement but had to pay only half the value in silver if the slave was blinded. The Code provided further detail for many procedures including those of veterinarians (“doctor of an ox or ass”).

Probably the most famous physician of all time and the founder of clinical medicine is Hippocrates (circa 460-360 B.C.E.) of Greek antiquity, the putative author of the *Corpus Hippocraticum.*^2^ Upon graduation from medical school, many modern physicians continue to take the Hippocratic Oath, the model of the ideal physician. Many historians question whether Hippocrates actually wrote this Oath or even the essays attributed to him. Some even question whether Hippocrates was a real person or was a composite created later by Greek and Roman scholars. Even in antiquity there were rules, policies, and regulations on how to behave as a physician.

Next to Hippocrates, Galen is probably the next most famous physician in history. His works and texts continued to be studied by medical students and scholars for hundreds of years after his death. When Galen ventured to Rome in 161 A.D. he was met with hostility by the medical establishment. For five years he was able to remain to practice medicine, lecture and conduct public discussions under the protection of the powerful Emperor Marcus Aurelius who named him the “first of physicians and philosophers”.^2^ Eventually, Galen left to return to Greece complaining that he had been driven out of Rome by the medical establishment who saw him as an interloper. Galen did subsequently return to Rome honoring a request from Marcus Aurelius. He remained for the rest of his life.

## Modern Regulations and Procedures for Licensing Physicians

The modern rules, policies and regulations governing the practice of medicine today are well established. In most jurisdictions – Canada, the United States, Europe – like the Code of Hammurabi, regulations govern nearly every aspect of the patient- physician relationship, clinical guidelines, best practices, evidence based medicine, fees, collegiality, and so forth. In Canada, for example, a number of organizations such as the Royal College of Physicians and Surgeons (RCPSC), the Medical Council of Canada (MCC), College of Family Physicians (CFP), and the provincial licensing authorities (e.g. College of Physicians and Surgeons of Ontario - CPSO), as well as doctor organizations such as the Canadian Medical Association (CMA), are all involved in governing the assessment, licensing, and behaviour of physicians in Canada.

Nearly all jurisdictions in the world nowadays require physicians to be university educated and earn an MD (medical doctor degree) or other approved degree (e.g., MBBS, MBChB). In many jurisdictions students who have earned a medical degree are required to undergo further postgraduate supervised clinical training in the United States called residency, also called a house officer or senior house officer in the United Kingdom and several Commonwealth countries. Depending on the medical specialty and jurisdiction, residency can be 1 to 6 years in duration. Once a doctor has passed all relevant examinations and qualifying procedures, the physician may be granted a license in a specified jurisdiction to practice medicine without direct supervision.

Doctors who have earned their degrees and qualifications from other jurisdictions and come to Canada, the United States, Britain and other places, are called international medical graduates are not considered to be legally qualified to practice medicine in that jurisdiction and must go through a series of assessments, re-education, residency and further examinations. This control about who can practice medicine in particular jurisdictions has always existed. Leonardo Fioravanti, a Renaissance physician who held a MD degree from the University of Bologna, a preeminent medical school of that time, ran into jurisdictional difficulties in his practice of medicine.^3^

## The Renaissance and the Case of Leonardo Fioravanti

At first frightened and then despondent, Fioravanti had been arrested and imprisoned by officers of the Public Health Board in Milan on the sketchy charge of not medicating in the accepted way. After eight days in prison, however, Fioravanti was becoming increasingly outraged by the indignity he was suffering. The Milanese physicians had been plotting against him since his arrival from Venice in 1572. They considered him an outsider, an alien and an unwelcome intruder. They finally were able to have him incarcerated.

Fioravanti was not a conventional medical charlatan hawking his nostrums in the piazza and then moving on. Nor was he a run-of-the-mill barber-surgeon. He had practiced medicine for years in Bologna, Rome, Sicily, Venice and Spain. He had a MD from the University of Bologna, had published several medical texts, had developed many medicines, and was a severe critic of much of conventional medical practice. The Milan physicians were not welcoming and considered him a foreign doctor.

A prison guard provided pen and paper for Fioravanti and in his most elegant and formal language, he addressed it to Milan’s public health minister from “Leonardo Fioravanti of Bologna, Doctor of Arts and Medicine, and Knight” (p. 7).^3^ He asked to be released from prison and to “medicate freely as a legitimate doctor”. A paid messenger delivered the letter to the Health Office located in the Piazza del Duomo.

The health minister, Niccolo Boldoni, was responsible for overseeing every aspect of medical practice in Milan, from examining midwives, barber-surgeons, and physicians, to collecting fees, imposing fines, inspecting apothecaries, and ruling on appeals. The letter from the Doctor and Knight, Leonardo Fioravanti, claimed that the Milan physicians were in a plot to stop him from providing care and cures to the sick of Milan. Moreover, he claimed that the Milan physicians were a menace to their patients and did more harm than good with quack treatments, poisonous medicines, and careless and arrogant behaviours. Fioravanti challenged the minister to provide 25 of the sickest patients to him and an equal number to Milan doctors that the minister selected and that he - Fioravanti - would cure his patients quicker and better than the other doctors. It is unlikely that this early clinical trial ever occurred as there is no historical record of it, but Boldoni and the Milan court set Fioravanti free.

## Patient Safety and Medical Errors

In 1999 - 500 years after Fioravanti’s indictment of Milan physicians - the Institute of Medicine of the National Academy of Sciences in the United States released the report, *To Err Is Human: Building a Safer Health System.*^4^ The report made the staggering claim that nearly 100,000 people in hospitals die annually in the United States as the result of medical mistakes. Subsequent commentators have suggested that this is an underestimate and the actual mortality rate is much higher. These claims triggered international discussion, concerns and controversies about patient injuries in health care. These errors are due to drug overdoses or interactions, misdiagnoses, botched surgeries, incorrect medications, and simple carelessness. Patient safety, a topic that had been little understood and even less discussed in health care systems, has become a public concern in most Western countries.

Notwithstanding its status as a mantra of modern medical practice, patient safety still requires investigation. Thousands of people are injured or die from medical errors and adverse events (incapacitation, serious injury or death) each year. Worldwide this figure may run into the millions. Leaders in the health care systems have emphasized the need to reduce medical errors as a high priority. Doctors, as main participants have been called upon to address the underlying systems causes of medical error and harm. Unfortunately, several studies have shown that even by 2007 more than half of hospital doctors surveyed^5^ had not even heard of the report, *To Err Is Human*.

It is not surprising then that few advances have been made in reducing medical errors and increasing patient safety in the past decade. A recent study of 464 major adult cardiac surgical cases at three hospitals resulted in 1,627 reports of problems and errors for an average of 3.5 and maximum of 26 per procedure. Nearly three-fourths of the cases (73.3%) had at least one recorded event. One-third (33.3%) of events occurred prior to the first incision, and 31.2% of events occurred while on bypass. About two-thirds (68.0%) of events were considered as minor in severity (e.g., delays and missing equipment), but a frightening percentage (32.0%) was considered major and included anastomotic problems (e.g., suturing vessels), pump failure, and drug errors. Many (30.9%) of the problems were never even discussed among the surgical team. A wide range of problems and errors occurs during the majority of cardiac surgery procedures.^6^ Cynics argue that the number of medical mistakes is much higher than is commonly accepted because most of the errors are buried with the patient.

The major factors underlying medical errors are thought to be system-based factors (miscommunication on the ward) as well as person factors: physician carelessness, ignorance, lack of professionalism, physician exhaustion and sleeplessness, physician arrogance, laziness, and poor self-assessment, particularly of personal limitations in medical skills.^7^,^8^ There is concern that the preferred tendency to put the emphasis on systems, but not holding individuals responsible for errors will weaken accountability for physician performance. Failure to hold individuals accountable may contribute significantly to risk of adverse events and may lead to a focus of patient safety away from the autonomous responsibility of physicians to a systems-based approach.

In the current issue of the *Canadian Medical Education Journal* we have included six major research contributions, two systematic review papers and three brief reports. Each of these addresses some aspect of patient safety, medical errors, practice guidelines and evidence based medicine.

## Major Research Contributions

Bass, Geddes, Wright, Coderre, Rikers and McLaughlin studied how experienced physicians benefit from analyzing initial diagnostic hypotheses. They began with the premise that most incorrect diagnoses involve at least one cognitive error, of which premature closure is the most prevalent. Thus Bass *et al* conducted an empirical study to evaluate the effect of analytic information processing on diagnostic performance of nephrologists and nephrology residents from the University of Calgary and Glasgow University. Participants were asked to diagnose ten nephrology cases. Participants were primed to use either hypothetico-deductive reasoning or scheme-inductive reasoning to analyze the remaining case data and generate a final diagnosis. The results indicated that both experienced nephrologists and nephrology residents can improve their performance by analyzing initial diagnostic hypotheses thus reducing the rate of misdiagnoses.

Documenting feedback during clinical supervision using field notes (FN) is a recommended competency-based evaluation strategy to improve communication. But what factors influence the intention to adopt FN during training? Lacasse, Douville, Desrosiers, Côté, Turcotte, and Légaré, used the theory of planned behaviour in a mixed-methods design, and employed clinical teachers (CT) and residents from two family medicine units to investigate the intention to adopt FN during training. They found that the intention to use FN were attitude, perceived behavioural control and normative beliefs. They concluded that the implementation of field notes should be preceded by interventions that target the identified salient beliefs to improve this competency-based evaluation strategy.

Does empathy towards patients in students change during medical school? What factors affect pre-clerkship changes in empathy? Sheikh, Carpenter and Wee recruited 12 students in their second year of medical school at Queen’s University to participate in semi-structured interviews conducted from an ethnographic perspective. Students reported both negative and positive changes in empathy. Negative changes included desensitization and focusing on the disease process, decreased ability to see things from patients’ perspectives, and routine responses in emotional situations. These changes occur due to time constraints, objective lessons in empathy, and a changing identity. Positive changes included an increased awareness of the impact of illness, and increased ability to read feelings. These changes result from increased exposure to patients, discussions surrounding the psychosocial impact of illness, and positive role models.

McKee, D’Eon, and Trinder analyzed the theory and pedagogical basis of the use of problem-based learning (PBL) for inter-professional education (IPE) in undergraduate health science education. They collected more than 1000 student surveys over 4 years that focused on components of usefulness, enjoyment and facilitator effectiveness. A retrospective self-assessment of learning was used for both content knowledge of palliative care and knowledge of the other professions participating in the module.

Medical students reported lower gains in knowledge than those in other programs. Scores were moderately high for usefulness and facilitator effectiveness. Scores for enjoyment were very high. McKee et al concluded that there is strong theoretical and empirical evidence that PBL is a useful method to deliver IPE for palliative care education.

Paslawski, Kearney and White addressed the factors that contribute to tutor participation in PBL in a medical training program, examining tutor recruitment and retention within the larger scope of teacher satisfaction and motivation in higher education. Semi structured interviews approximately one hour in length were conducted with 14 people - 11 who had tutored in PBL and 3 faculty members who had chosen not to participate in PBL. Thematic analysis was employed as the framework for analysis of the data. Seven factors were identified that affects the recruitment and retention of tutors in the undergraduate medical education program.

Ma, Wishart, Kaminska, McLaughlin, Weeks, Lautner, Baxter, and Wright addressed the question, “How can teaching physical examinations at the undergraduate level be improved”? They studied the use of ultrasonography, a method increasingly used for teaching physical examination in medical schools.

Surveying the opinions of involved educators, they identified potentially useful aspects ultrasonography: measuring the size of the abdominal aorta, identifying the presence/absence of ascites, identifying the presence/absence of pleural effusions, and measuring the size of the bladder. Examinations thought to be potentially most harmful included: identifying the presence/absence of intrauterine pregnancy, measuring the size of the abdominal aorta, and identifying the presence/absence of pericardial effusion. Ma et al caution that when initiating an ultrasound curriculum for physical examinations, educators should weigh the risks and benefits of examinations chosen.

## Systematic Reviews

In the first of two systematic reviews, Al Alawi, Al Ansari, Raees and Al Khalifa, focused on the use of multisource feedback to assess pediatricians. Having searched the electronic databases EMBASE, PsycINFO, MEDLINE, PUBMED, and CINAHL they identified six studies that met the inclusion criteria. Al Alawi et al found high internal consistency reliability in five studies (α ≥ 0.95) and generalizability in two studies (Ep^2^ ≥ 0.78). Additionally evidence for content, criterion-related and construct validity was reported in all 6 studies. They concluded that multisource feedback is a feasible, reliable, and valid method to assess key competencies such as communication skills, interpersonal skills, collegiality, and medical expertise.

The second systematic review of educational resources for teaching patient handover skills to resident physicians and other healthcare professionals was done by Masterson, Richdeep, Turner, Shrichand, and Giuliani. As the transfer of patient care is a time of heightened risk to patients, it is important to identify effective training models for handover skills. A number of such studies have now been published. Masterson et al found that physicians, residents and other healthcare practitioners should receive training in handover skills to improve patient care and thus reduce the risk of medical errors.

## Brief Reports

In the first of three brief reports, Thomson, Harley, Cave and Clandinin studied the enhancement of medical student performance through narrative reflective practice (NPR). This process putatively helps medical students become better listeners. Employing 139 3^rd^-year University of Alberta medical students from the same class, they found that the group receiving NRP training scored higher (4.7%) on multiple-choice question exams (MCQs) but not on objective structured clinical examinations (OSCEs) or on subjective clinical evaluations (SCEs). Two weeks NRP exposure produced an increase in students’ MCQ scores; perhaps longer periods of NRP exposure may also increase the OSCE and SCE scores.

The second brief report focused on the Triple C curriculum for preparing residents for family practice. In this study, Lee, McMillan, Hiller and O’Brien focused on the impact of Triple C competency-based curriculum on the preparation of residents for family practice. Residents perceived themselves as prepared to engage in most practice areas and their intentions to engage in various practice domains were positively correlated to their ratings of preparedness. Residents perceived this program as comprehensive and relevant to their development as a family physician and they perceived a high degree of encouragement for inter-professional practice. These results provide some preliminary evidence that an integrated competency-based curriculum, with an emphasis on inter-professional practice, has the potential to effectively prepare residents for practice in family medicine.

In their brief report Kassam, Donnon, Cowan and Todesco described two ways to assess the *Scholar* CanMEDS role using a modified OSCE format where two stations consisted of 1) critically appraising an article, and 2) critiquing an abstract. Sixty-three residents completed the CanMEDS In-Training Exam including the two *Scholar* stations. There were no significant differences between the global scores of the *Scholar* stations showing that the overall knowledge and effort of the residents was similar across both stations (3.8 vs. 3.5, *p* = 0.13). No significant differences between senior residents and junior residents were detected or between internal medicine residents and non-internal medicine residents.

In this issue the major research contributions, systematic review papers, and brief reports each address some variant of improving medical practice and therefore improving patient care and safety. In addition we are publishing commentaries and letters to the editor and two brief essays by students on the future of medical and health care education.
